# Unravelling High Nuclear Genomic Similarity and Mitochondria Linked Epigenetic Divergence in SCNT Derived Buffalo Clones via Long-Read Nanopore Genome Sequencing

**DOI:** 10.3390/ijms26188836

**Published:** 2025-09-11

**Authors:** Meeti Punetha, Dharmendra Kumar, Satish Kumar, Bhavya Maggo, Priya Dahiya, Pradeep Kumar, Rakesh K. Sharma, Yash Pal, Prem S. Yadav

**Affiliations:** 1 Animal Physiology and Reproduction Division, ICAR-Central Institute for Research on Buffaloes, Hisar 125001, Haryana, India; meetipunetha283@gmail.com (M.P.);; 2 Animal Genetics and Breeding, ICAR-National Research Centre on Pig, Rani, Guwahati 781131, Assam, India; hilsa.satis2007@gmail.com

**Keywords:** cloned buffalo, methylome, epigenetic, SCNT, structural variants

## Abstract

Somatic cell nuclear transfer (SCNT) holds promise for animal cloning but remains limited by low efficiency and phenotypic abnormalities, often attributed to incomplete nuclear reprogramming. This study presents an integrative genomic and epigenomic analysis of cloned buffaloes and their respective donors using long-read Oxford Nanopore sequencing. Our results showed a high degree of genomic similarity between clones and donors, with most variations located in non-coding regions and structural variants (SV) distributions highly correlated at the chromosomal level. Gene and protein level overlap of SV-affected loci revealed 70.9–73.3% gene-level and 69.7–72.5% protein-level similarity. Despite this genetic similarity, DNA methylation analysis identified differentially methylated regions (DMRs), particularly in intergenic and promoter regions. Clones exhibited slightly lower CpG methylation than the donors. The DMRs in donor vs. clone comparisons indicated higher hypomethylated regions than hypermethylated regions. Functional enrichment of DMR-associated genes highlighted pathways linked to mitochondrial function, oxidative phosphorylation, and reproductive processes. Although clones showed moderate genome-wide methylation correlation with donors, key differences in methylation suggest incomplete epigenetic reprogramming. Despite these epigenetic differences, all clones were phenotypically normal and healthy into adulthood. This study offers the first comprehensive SV and methylome profile of SCNT-derived buffaloes and emphasizes the role of epigenetic mechanisms in clone development and health, providing valuable insights to enhance cloning efficiency.

## 1. Introduction

Somatic cell nuclear transfer (SCNT), widely known as cloning, is a sophisticated biotechnological method that enables the generation of genetically identical organisms by transferring the nucleus of a diploid somatic cell into an enucleated oocyte. This process results in the formation of a cytohybrid (cytoplast + karyoplast), which, upon activation, initiates reprogramming of the somatic nucleus to a totipotent state, allowing for embryonic development and the potential production of live cloned offspring [1,2]. SCNT has diverse applications in animal breeding, regenerative medicine, and biomedical research. However, the success of nuclear reprogramming, a critical determinant of cloning efficiency, is highly influenced by the molecular and cytoplasmic milieu of the recipient oocyte at the time of fusion [3].

Although clones are intended to be genetically and phenotypically identical to their donors, they often display anomalies absent in the donor, such as underweight, and cardiac and immune dysfunctions, likely stemming from incomplete or aberrant nuclear reprogramming [4,5,6,7,8,9]. Nuclear reprogramming involves extensive epigenetic remodeling, whereby the somatic cell’s epigenetic marks are erased and re-established to match the embryonic totipotent state. This process requires the reactivation of approximately 10,000–12,000 genes essential for embryogenesis [10,11]. Aberrations in this process are widely recognized as a primary cause of the phenotypic discrepancies and low viability observed in cloned animals.

DNA methylation, one of the most critical epigenetic modifications, plays a central role in nuclear reprogramming during SCNT. It regulates genomic imprinting, X-chromosome inactivation, genome stability, and suppression of retrotransposons, among other functions. Traditionally, DNA methylation analysis relied on bisulfite conversion and short-read sequencing, which is limited by read length and potential DNA degradation [12]. Recent advances in third-generation sequencing technologies, such as Oxford Nanopore Technologies (ONT) and PacBio’s single-molecule real-time (SMRT) sequencing, have enabled direct, real-time detection of nucleotide modifications at a genome-wide scale without bisulfite treatment. Nanopore sequencing identifies epigenetic marks by detecting electrical current changes (termed “squiggles”) as DNA passes through the nanopores, providing both sequence and methylation data simultaneously [13,14,15,16].

A PromethION run can provide whole-genome data from genomic DNA at an average depth ranging from ×15 to ×30, which could be exploited for variant calling. Beyond single nucleotide variants (SNVs), assessing structural variants (SVs)—including insertions, deletions, duplications, inversions, and translocations ≥50 bp—is critical for evaluating genomic integrity in cloned animals. SVs are major contributors to genetic and phenotypic diversity and are implicated in evolutionary processes and various diseases [17,18,19,20,21,22,23,24,25,26]. Although SCNT aims to create genetically identical animals, assessing the structural genomic similarity between donor and clone is essential.

The clones used in the present study were produced by our group using the Hand-made Cloning (HMC) method, as previously described by Yadav et al. (2024) [1] and Selokar et al. (2019) [2]. This technique has been successfully employed to generate viable cloned embryos and offspring in buffalo. Post-natal growth, hematology, telomere length, and semen attributes of these clones have already been studied and were found to be normal [1]. However, the comprehensive genome-wide analysis to evaluate genetic and epigenetic integrity in cloned buffaloes compared to their respective donors has not been studied until now. Therefore, in the present study we examined single nucleotide polymorphisms (SNPs), small insertions and deletions (InDels), and SVs to assess genomic similarity using long-read Nanopore sequencing. In parallel, we performed CpG methylation profiling to identify differentially methylated regions and genes between clones and donors. Functional annotation and gene ontology enrichment were further employed to understand the potential biological significance of these epigenetic alterations. This integrative approach provides novel insights into the genomic and epigenomic landscape of cloned animals and highlights the potential role of epigenetic reprogramming in clone development.

## 2. Results

### 2.1. DNA Methylation Mapping and Patterns

The results of the Nanopore PromethION sequencing to assess DNA methylation are shown in Table 1. The sequencing coverage obtained for raw Nanopore data ranged from 16.6X to 19.7X, generating ~43 to 51 GB of long-read data across four samples. Around 99% of the reads generated had a minimum quality score of 10. In the mapping step, 99% of reads were aligned to the buffaloes’ reference genome (GCF_019923935.1). These results confirm the high quality of sequencing data, which is essential for reliable downstream bioinformatics analyses. The whole genome sequencing (WGS) data has been submitted to NCBI under the accession number (PRJNA978520), ensuring public access for further research and analysis.

### 2.2. Variant Analysis

**Figure 1:**
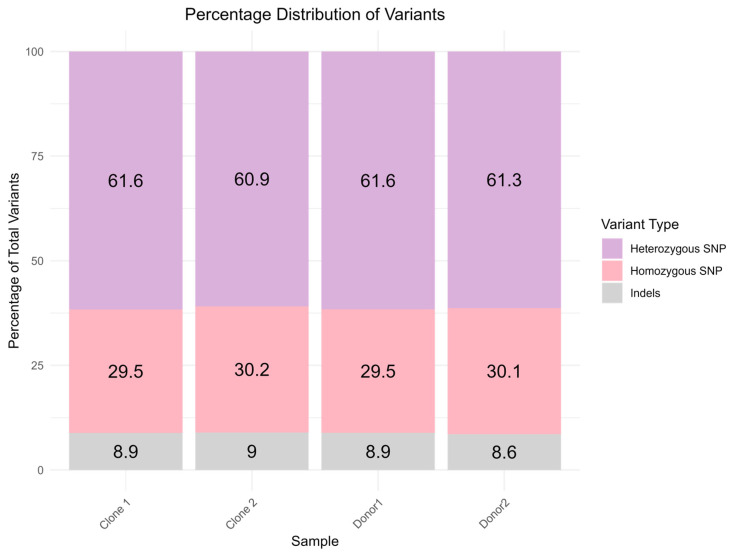
Bar plot showing the percentage distribution of variants.

WGS data were aligned to the *Bubalus bubalis* reference genome to identify the variants in the form of SNPs and INDELs. Further, the identified variants were also annotated for predicting the effects of genetic variants on genes and proteins. The total number of SNPs and other variants along with their distribution is given in Figure 1 (Supplementary Files S1–S4).

Most variants were located in intronic regions, followed by intergenic, upstream, and downstream regions. A smaller proportion of variants were found in exonic regions (Table 2). The nonsynonymous SNPs were about 1.6 to 2.9% of the total SNPs predicted.

### 2.3. Structural Variant (SV) Analysis

Whole-genome SV analysis was performed on two donor-clone pairs: donor 1 and clone 1, as well as donor 2 and clone 2. Initial SV calls identified 130,926 and 131,313 variants in donor 1 and clone 1, respectively, and 137,597 and 141,824 in donor 2 and clone 2, respectively. After stringent filtering, a total of 61,266 (donor 1), 62,687 (clone 1), 64,335 (donor 2), and 71,820 (clone 2) high-confidence SVs were retained for subsequent analysis.

The SVs predominantly consisted of deletions (DEL, ~60%) and insertions (INS, ~38%), with minor contributions from breakends (BND, ~1.5%), duplications (DUP, ~0.3%), and inversions (INV, ~0.1%) (Table 3). The distribution of variant types was consistent between donors and their respective clones, suggesting low structural variation.

### 2.4. SV Overlap Between Donors and Clones

**Figure 2:**
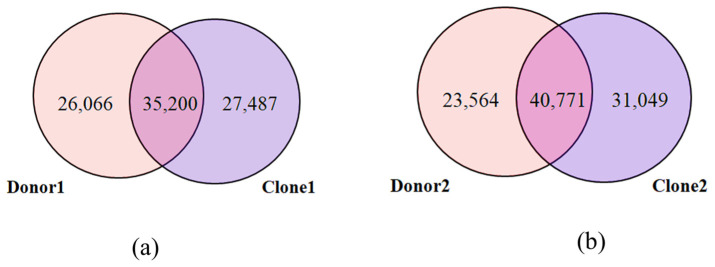
Venn diagram illustrating the overlap and uniqueness of structural variants (SVs) between (**a**) donor 1 and clone 1, and (**b**) donor 2 and clone 2.

Direct overlap analysis revealed that 35,200 SVs were shared between donor 1 and clone 1, with 26,066 and 27,487 SVs unique to donor 1 and clone 1, respectively. Similarly, donor 2 and clone 2 shared 40,771 SVs, while 23,564 and 31,049 SVs were unique to donor 2 and clone 2, respectively (Figure 2). These results demonstrate substantial retention of donor SVs in clones, while also suggesting that the SVs arose either during development of the calf or in the cell from which they were derived. Fisher’s exact test showed that the overlap in SVs is highly statistically significant (*p* < 0.001), suggesting that SV sharing is non-random and driven by clonal inheritance.

### 2.5. Functional Annotation of Affected Genomic Regions

**Figure 3:**
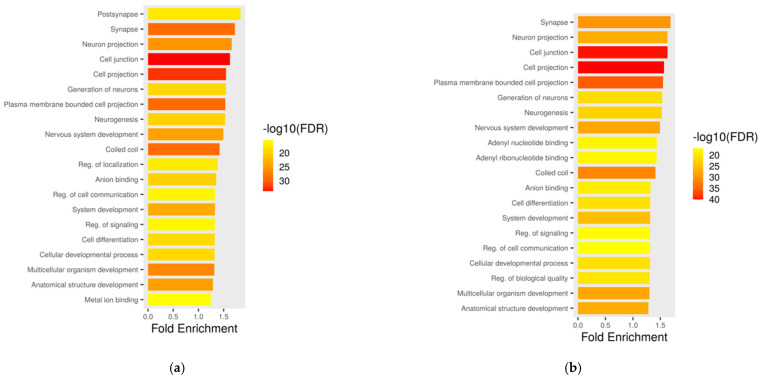
Functional annotation plot of SVs located on genes shared between (**a**) donor 1 and clone 1, and (**b**) donor 2 and clone 2.

Annotation of SVs revealed that the majority resided in protein-coding regions (86.25% in donor 1, 85.76% in clone 1, 86.76% in donor 2, and 85.98% in clone 2) (Table 4). Long non-coding RNAs (lncRNAs, ~11–12%) and pseudogenes (~2%) were also consistently affected across samples. Minor fractions were found in other biotypes (C regions, rRNA, tRNA, etc.). The distribution of gene biotypes affected by SVs was highly consistent between donors and their respective clones, indicating that SVs predominantly affect similar functional genomics regions. To further explore the biological relevance of the affected genes, functional enrichment analysis was performed on genes located in SV regions shared between donors and clones. Several significantly enriched gene ontology (GO) biological processes were identified, including cell junction (GO:0030054), cell projection (GO:0042995), synapse (GO:0045202), neuron projection (GO:0043005), and system development (GO:0048731). KEGG pathway analysis of shared genes in the donor–clone pair revealed involvement in the MAPK signaling pathway, calcium signaling pathway, oxytocin signaling pathway, and Rap1 signaling pathway with additional involvement in regulation of the actin cytoskeleton. To aid visualization, a functional annotation bar plot has been included (Figure 3) summarizing the major enriched pathways and biological processes, thereby enhancing the understanding of their biological significance.

### 2.6. Gene- and Protein-Level Similarity Between Donor–Clone Pairs

Gene- and protein-level overlap of SV-affected loci was evaluated, revealing a 70.95% similarity between donor 1 and clone 1 and a 73.32% similarity between donor 2 and clone 2 at the gene level. At the protein level, the similarity was 69.69% for donor 1 vs. clone 1 and 72.52% for donor 2 vs. clone 2.

### 2.7. Chromosomal Distribution of SVs

SV distributions at the chromosomal level were highly correlated between donors and clones, with Pearson correlation coefficients of 0.997 for donor 1 vs. clone 1 and 0.998 for donor 2 vs. clone 2. The high correlation suggests that the donors and clones share a strong genetic background indicating a common origin, population, or evolutionary history.

### 2.8. Characterization of Methylated Regions

**Figure 4:**
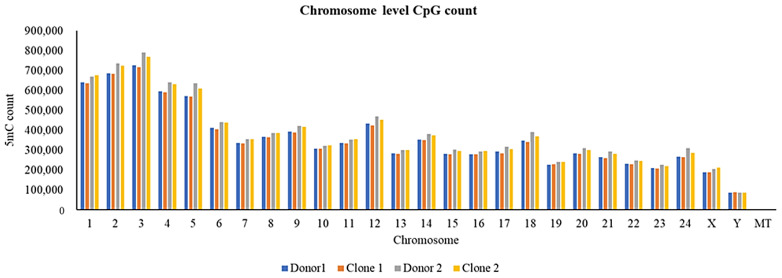
Genome-level CpG methylation distribution.

**Figure 5:**
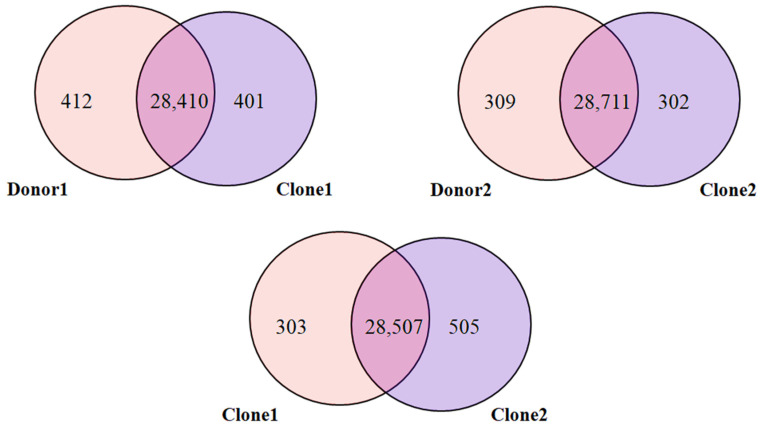
Venn diagram of overlapping methylated genes in clones versus their respective donors.

The aggregated CpG methylation count, having a minimum read depth of five, ranged from 9.3 million to 10.1 million (Table 5). CpG methylation analysis was conducted, and feature annotations were mapped for precise localization of methylation patterns relative to genomic features. Around 60% of the predicted CpG methylation sites were mapped to gene regions in the reference genome. The distribution of CpG sites across various chromosomes of donor 1, clone 1, donor 2 and clone 2 is illustrated in Figure 4. Further, DNA methylation of CpG islands within genic regions on each chromosome was determined. A total of 29,104, 29,089, 29,307, and 29,306 methylated genes were identified for donor 1, clone 1, clone 2 and donor 2, respectively. Venn diagram analysis revealed that 28,410, 28,711, and 28,507 methylated genes overlapped among donor 1 vs. clone 1 (Figure 5), donor 2 vs. clone 2 and clone 1 vs. clone 2, respectively. Unique methylated genes identified in the clones included natriuretic peptide receptor 2 (*NPR2*), Endothelin 1 (*EDN1*), natriuretic peptide C (*NPPC*), Endothelin receptor type A (*EDNRA*), progesterone receptor membrane component 1 (*PGRMC1*), spermatid maturation 1 (*SPEM1*), apolipoprotein E (*APOE*), XK related 6 (*XKR6*), and ATPase phospholipid transporting 8B1 (*ATP8B1*).

### 2.9. DNA Methylation Conservation Between the Donor and Clone Bulls

**Figure 6:**
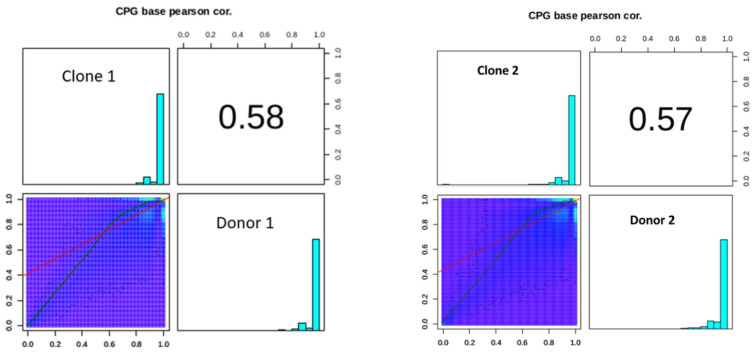
The correlation analysis of donor and clone. In the histogram of methylation level in the figure, the abscissa is the methylation level and the ordinate is the proportion of the corresponding methylation level; the value in the figure is the correlation coefficient of two samples.

To unravel the DNA methylation conservation patterns among individuals, we correlated the DNA methylation levels between the donor and clone bulls in different chromosomes. Our results showed that the DNA methylation of both the donor and clone bulls was 58% correlated with each other on a genome-wide level (Pearson’s r = 0.58) (Figure 6), implying that our datasets had accurate callings. A correlation coefficient of 0.58 indicates a moderate positive correlation between the methylation levels of the donor and the clone, which indicates that the regions that are highly methylated in the donor tend to be relatively highly methylated in the clone as well, and vice versa.

### 2.10. Differential Methylation of the Hypermethylated and Hypomethylated Island Annotation Between the Clone and Donor

**Figure 7:**
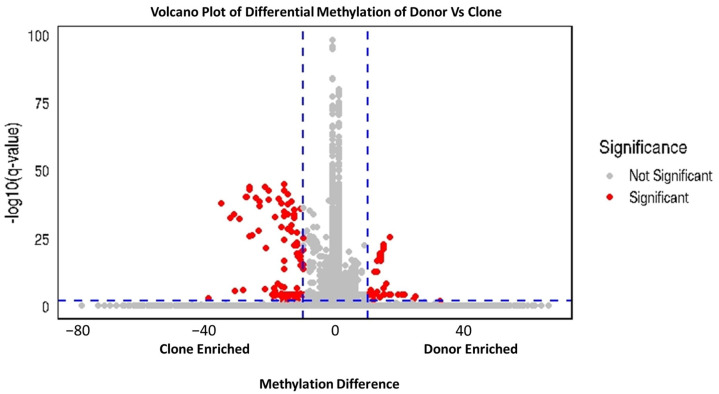
Volcano plot representing q-value and methylation difference in all CpG sites compared between donors and clones. Red dots represent significantly differentially methylated CpG sites (methylation difference >10%, q < 0.01) while grey dots represent nonsignificant differential methylated CpG sites Blue dashed line is demarking the significant and nonsignificant differential methylated CpG sites.

**Figure 8:**
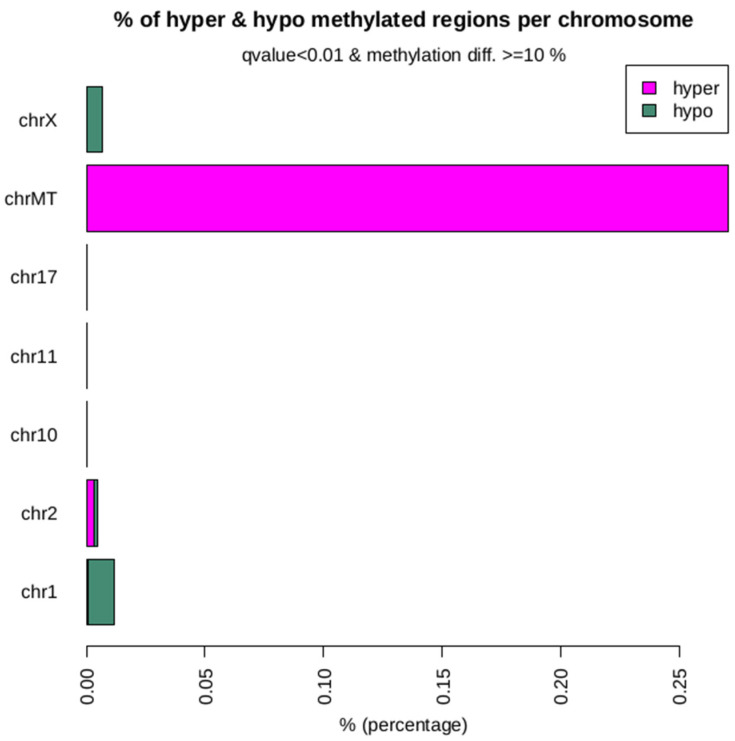
Percentages of significant hypomethylated and hypermethylated DMGs in different chromosomes between donor 2 and clone 2.

The differential methylation analysis was carried out between donor and clone at a threshold of 10% difference in methylation level and q-value of 0.01 for significant hyper and hypomethylated bases. We identified 7,743,726 and 8,821,206 DMRs between donor 1 vs. clone1 and donor 2 vs. clone 2 bulls by applying a threshold of ≥10% methylation difference and q < 0.01, respectively, of which 53 regions were hypermethylated and 115 regions were hypomethylated (Table 6; Figure 7; Supplementary Files S5 and S6). Among hypomethylated CpG islands, 89.47% were located in intergenic regions, 5% in promoter regions, and 2.63% each at the transcription termination site (TTS) and in the exons, whereas 100% of hypermethylated CpG Islands were found in intergenic regions. Chromosome preference analysis showed that chromosomes 1, 2, 10, 11, 17, MT and X were enriched in DMRs (Figure 8).

### 2.11. GO Enrichment and Pathway Analyses of Differentially Methylated Genes

**Figure 9:**
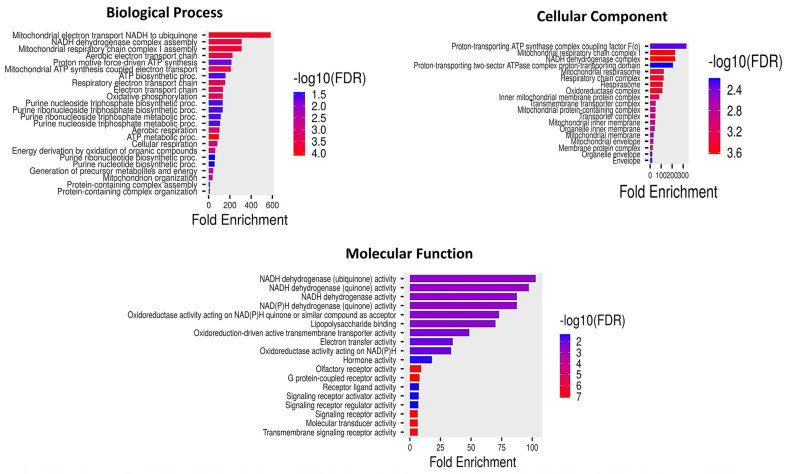
Gene ontology analyses of the differentially methylated genes between clones and their respective donors.

To assess if the genes associated with differential methylation were enriched in some biological processes or pathways, we conducted gene ontology and pathway analyses using DMGs. The details were retrieved from the Uniport database, and pathway information was retrieved from the KEGG pathway database. GO enrichment analysis of significantly enriched DMG between donors and clones was categorized into biological processes, cellular components and molecular functions (Figure 9). Among the top DMG-enriched biological processes, the following processes were relevant: mitochondrial electron transport (GO:0006120, GO:0019646, GO:0009060, GO:0045333, GO:0015980, GO:0006091); oxidative phosphorylation (GO:0006119, GO:0022900, GO:0042775, GO:0042773, GO:0045333); the cellular component relevant to mitochondrial respiratory chain complex (GO:0045271, GO:0098803, GO:0098796, GO:0070469, GO:1902495, GO:1990351, GO:0030964), mitochondrial inner membrane (GO:0005743, GO:0019866, GO:0031966); and molecular functions related to NADH dehydrogenase (ubiquinone) activity [GO:0008137], G protein-coupled receptor signaling (GO:0007186), signaling transduction (GO:0007165). The KEGG analysis identified genes NADH dehydrogenase subunit 1 (*ND1*), NADH dehydrogenase subunit 2 (*ND2*), and ATP synthase F0 subunit 6 (*ATP6*), which are associated with oxidative phosphorylation and neurodegeneration pathways. Other genes, such as olfactory receptor family 6 subfamily Z member 9 (*OR6Z9*), olfactory receptor family 9 subfamily K member 15 (*OR9K15)*, and olfactory receptor family 4 subfamily C member 1F (*OR4C1F*) were enriched with olfactory transduction pathway.

## 3. Discussion

SCNT is a cloning technique in which the nucleus of a differentiated somatic cell (karyoplast) is transferred into an enucleated oocyte (cytoplast) to generate a cloned embryo subsequently transferred into a synchronized female to produce a cloned calf. The resulting clone inherits its nuclear genome from the donor cell but retains mitochondrial DNA from the oocyte, leading to formation of genetic chimerism between nuclear and cytoplasmic components. This mismatch can result in mitochondrial–nuclear incompatibilities that may disrupt cellular energy metabolism, impair embryonic development, and contribute to the low efficiency and abnormal phenotypes often observed in SCNT-derived animals [27,28]. Despite being genetically identical at the nuclear level, clones frequently display developmental anomalies, reduced survival rates, and altered physiology, largely attributed to incomplete or aberrant epigenetic reprogramming and potential genomic instability introduced during the cloning process [10].

Most studies assessing genomic similarity between clones and their nuclear donors have focused on small-scale variants such as single nucleotide polymorphisms (SNPs) and small InDels. However, SVs viz. insertions, deletions, duplications, inversions, and translocations ≥50 bp represent a major source of genomic variation and can significantly influence gene function and genome architecture [17,18,19]. For the first time, we performed a comprehensive genome-wide assessment of donor–clone similarity using WGS-based SV analysis combined with DNA methylation profiling. This dual approach provides critical insight into both the genomic integrity and epigenetic fidelity of SCNT-derived animals. The SV analysis enabled the detection of large-scale genomic alterations potentially introduced during donor cell culture, nuclear reprogramming, or early embryonic development. In parallel, CpG methylation profiling revealed DMRs between clones and their respective donors, offering a deeper understanding of epigenetic reprogramming during SCNT. Together, these findings contribute valuable knowledge toward improving cloning efficiency, ensuring genomic stability, and safeguarding the developmental health and normalcy of cloned offspring.

Most of our knowledge on speciation genomics is based on single-nucleotide polymorphisms (SNPs), mainly because such variants are easily accessible with short-read sequencing [29,30]. However, genomes also vary in structure with loss, gain or rearrangement of sequences between individuals and between species. Such SVs are now recognized to be ubiquitous and to affect a larger fraction of the genomes than SNPs [31,32]. SVs may also have large phenotypic effects, may impact recombination, and may be involved in speciation [33,34,35]. Our whole-genome SV analysis revealed that deletions and insertions were the most common variant types (~98% combined), and the distribution of SV types was consistent between donors and their clones. Our analyses of SVs in buffalo genomes provide an overview of the genomic landscape of SVs between donor and clone. SNPs and INDELs were identified using Clair3 and functionally annotated with SnpEff. We observed a high number of shared variants between donors and clones, with nonsynonymous SNPs accounting for approximately 1.6–2.9% of the total SNPs, most of which were located in intronic and intergenic regions, suggesting that the majority of variants are unlikely to disrupt protein function.

However, despite the high sequence and structural similarity between donor and clone genomes, we also conducted a comprehensive analysis of DNA methylation to evaluate epigenetic concordance between the pairs. DNA methylation is an important epigenetic modification that plays a critical role in mammalian development. It involves the transfer of a methyl group onto the C5 position of the cytosine to form 5-methylcytosine. DNA methylation patterns in SCNT embryos by immunofluorescence staining suggested that DNA methylation in donor cells is not completely reprogrammed in SCNT embryos [36,37]. The epigenetic mechanisms in pre-implantation and nuclear-transferred embryos have been extensively studied, with findings that have contributed to enhancing cloning efficiency [38,39,40,41]. Studies specifically comparing the epigenetic variations between cloned buffaloes and their donor animals are limited, as most epigenetic research in livestock cloning has focused on early developmental stages, such as pre-implantation embryos and fetal development. Therefore, the current study aimed to identify potential epigenetic variations between adult cloned buffaloes produced through SCNT and their respective donors to provide valuable insights into the long-term effects of SCNT on epigenetic stability in this species, which could impact traits like health and reproductive performance.

The Nanopore PromethION sequencing data of clones and their respective donors were generated, and, after comparing the dynamics of DNA methylation, we found that the global DNA methylation levels in clones and donors were not of much difference. In addition, the methylation rate of CpG among different chromosomes showed some variations. To evaluate the influence of aberrant DNA methylation levels, we first defined the DNA-methylation-affected genes between clones and donors. A total of 703 methylated genes were found to be unique in clones as compared to donors. Functional analysis of these genes revealed that they were largely enriched in biological processes related to meiotic cell cycle processes during oocyte maturation, neuroactive ligand receptor interaction, and spermatid maturation. These processes are critical for fertility, reproduction, and proper embryonic development. It has been suggested that the SCNT technique, which involves transferring nuclei from differentiated somatic cells into enucleated oocytes, may disrupt normal reprogramming processes during embryonic development, potentially resulting in impaired gene regulation [42].

To investigate the conservation patterns of DNA methylation among individuals, the correlation of DNA methylation levels between the donor and clone bulls across different chromosomes was analyzed. Our findings revealed a genome-wide DNA methylation correlation of 58% between the donor and clone bulls. While there is a noticeable relationship, the correlation is moderate, not very strong. Other factors might be influencing the methylation levels, and the methylation patterns in the donor and clone are not perfectly aligned. The methylation patterns between the donor and the clone are somewhat similar but not identical. It points to both the retention of some epigenetic marks from the donor and the occurrence of differences that might be due to various biological, environmental, or technical factors [43,44]. To further study the underlying molecular mechanism of DNA methylation, we performed DMR analysis in clones and donors using Methylkit. The identified DMRs were divided into hyper-DMRs and hypo-DMRs by comparing DNA methylation levels of the DMRs in clones and donors (% difference of 10 with q-value threshold of 0.01). Based on our methylome analysis, the cloned group displayed more hypomethylation than hypermethylation in the whole genome compared to their donor, and this result is similar to other reports [45,46]. Furthermore, we found that the majority of methylation occurs in the intergenic region using the Homer tool based on reference genomic annotation data. A total of 2.63% of hypermethylated CpG islands were found at the TTS and exon of clone buffalo which suggested that methylation status in exon regions of genes could potentially risk abnormal gene expression, thus leading to changes in the cloned phenotype. In addition, we found that the methylation-affected genes were highly enriched for mitochondrial function, energy metabolism, and cellular signaling. Accumulating evidence suggests that incompatibility between the nucleus and mitochondrial DNA (mtDNA), caused by the mixing of two or three different mtDNA genotypes (heteroplasmy) in cloned embryos, may affect the expression of oxidative phosphorylation (OXPHOS) genes, leading to varying efficiencies in ATP production [47,48,49]. Many studies have shown that altered mitochondrial DNA causes dysfunction, which is responsible for triggering aging and degenerative diseases [50]. These pathways are vital for maintaining cellular homeostasis, and disruptions in their regulation can lead to a wide range of pathologies, including metabolic disorders, neurodegenerative diseases, and cancers. Despite observing aberrant methylation patterns in mitochondrial function, the clones under this study are healthy and have survived and thrived reaching 6 and 10 years of age. These clones are healthy, and their growth, aging, and fertility parameters have already been studied, demonstrating that they are healthy and indistinguishable from their non-cloned counterparts [1].

## 4. Materials and Methods

All of the procedures in these experiments were performed using minimally invasive techniques, strictly following humane endpoint guidelines to ensure the animals’ welfare. The project was approved by the institute’s Animal Ethics Committee under approval number IAEC-CIRB/19–20/A/006 Date of approval 1 January 2019, in accordance with established ethical standards for animal care and use.

### 4.1. Animals and Sampling

The clones used in the study, 4998 (Hisar Gaurav) and E-265, were males and produced via HMC method in which the somatic cells of elite bull 4354 and M29 were used as donors. At the time of manuscript writing, the clones were of 10 years and 6 years of age and were healthy, whereas the donors 4354 and M29 were of 14 years and 20 years of age, respectively.

### 4.2. DNA Extraction and Quality Control

Genomic DNA was extracted from whole blood samples from two donors (Donor 1: M29; Donor 2: 4354) and their clones produced earlier by our team [1,2] (Clone 1: E-265; Clone 2: 4998) using the Blood and Cell Culture DNA Mini Kit (Cat. No: 13323, Qiagen, Hildan, Germany). DNA concentration and purity were assessed using a NanoDrop Spectrophotometer (Thermo Scientific, Model 2000, Wilmington, DE, USA). DNA integrity was confirmed by agarose gel electrophoresis, and accurate concentration was quantified using the Qubit dsDNA HS Assay Kit (Thermo Fisher Scientific, Hillsboro, OR, USA). The DNA samples with good quality and purity were used for the downstream analysis.

### 4.3. Library Preparation

High molecular weight DNA was fragmented to appropriate sizes by physical shearing, and fragment distribution was evaluated using Genomic DNA ScreenTape (Agilent Technologies, Santa Clara, CA, USA). End-repair and A-tailing of DNA fragments were carried out using the NEB Next Ultra II End-Repair Kit (New England Biolabs, Ipswich, MA, USA). Barcoding of DNA samples was performed using Blunt/TA Ligase Master Mix (New England Biolabs, Ipswich, MA, USA). Equimolar concentrations of barcoded samples were pooled.

Sequencing adapters (SQK-LSK114.24) (Oxford Nanopore Technologies, Oxford, UK) were ligated to double-stranded DNA fragments using NEB Quick T4 DNA Ligase (New England Biolabs, Ipswich, MA, USA). Purification of the prepared library was performed using AMPure XP beads (Beckman Coulter, Brea, CA, USA). The final library was subjected to quality control before sequencing.

### 4.4. Nanopore Sequencing

The QC-checked libraries were quantified using the Qubit Fluorometer and loaded onto PromethION Flow Cells (FLO-PRO114M) for sequencing using the Oxford Nanopore Technologies PromethION P24 platform (Oxford Nanopore Technologies, Oxford, UK). Sequencing was conducted according to the manufacturer’s protocol.

### 4.5. Bioinformatics and Data Analysis

High-throughput Nanopore sequencing data from four buffalo samples were processed through a comprehensive bioinformatics workflow to ensure accurate variant detection, methylation analysis, and genome annotation.

#### 4.5.1. Base Calling and Quality Assessment

Raw Nanopore reads were base called using Guppy (v.6.4.6+ae70e8f) in high-accuracy mode to generate FASTQ files. Quality control of sequencing data was performed using Commander-(v.2.0.2), ensuring that over 99% of reads achieved a minimum quality score of Q10.

#### 4.5.2. Read Alignment and Reference Mapping

Reads were aligned to the *Bubalus bubalis* reference genome (GCF_019923935.1) using Minimap2 (v2.24-r1122), generating sorted BAM files for each sample.

#### 4.5.3. DNA Methylation Analysis

To assess DNA methylation (5mC) at CpG sites, modified base information was extracted from aligned reads. Methylation profiles were converted to BED format using modbam2bed (v.0.5.2), and genomic feature annotations were applied using BEDTools (Bedtools-v2.30.0). Differentially methylated regions (DMRs) between donor and clone samples were identified using the MethylKit R package (https://github.com/al2na/methylKit, accessed on 5 August 2025), with criteria set at ≥10% methylation difference and q-value < 0.01. Gene annotation for DMRs was performed using HOMER to identify affected functional regions.

#### 4.5.4. Variant Calling and Annotation

Single nucleotide polymorphisms (SNPs) and small insertions/deletions (INDELs) were detected using Clair3 (v.1.0.0), a deep-learning-based variant caller optimized for long-read data. Detected variants were annotated using SnpEff (v.3.3h), providing functional categorization of coding and non-coding mutations.

#### 4.5.5. Structural Variant Detection

Structural variants (SVs), including deletions, insertions, duplications, inversions, and breakends, were identified using a custom pipeline implemented via standard Linux command-line tools. High-confidence SVs were filtered and validated using GATK (v.4.6.1.0). Annotation of SVs and their genomic context was carried out in R (v.4.5.0) using packages including Genomic Ranges (v.1.60.0), r track layer (v.1.68.0), Annotation Hub (v.3.16.1), and org.Bt.eg.db (v.3.21.0), enabling precise mapping of SVs to functional genomics elements such as genes, exons, and regulatory regions.

#### 4.5.6. Comparative Analysis of Donor and Clone Samples

To evaluate the genetic similarity between donor–clone pairs, SV overlap was assessed using Jaccard index analysis, while gene-level concordance was calculated based on shared affected loci. Chromosome-level SV distribution correlation was quantified using Pearson’s correlation coefficient.

#### 4.5.7. Gene Ontology and Pathway Enrichment

Genes associated with differential methylation were subjected to gene ontology (GO) and KEGG pathway enrichment analysis. Functional categories were retrieved from the UniProt database, and pathway associations were mapped using the KEGG resource.

## 5. Conclusions

Our comparative analysis reveals that SCNT preserves high genomic integrity between donor and clone, with SVs and SNPs largely shared and located in non-coding regions, indicating minimal disruption in protein function. The current findings underscore the importance of exploring mitochondrial pathways and understanding the impact of methylation changes on cellular function. Additionally, they serve as an invaluable resource for exploring the epigenetic and environmental influences on the wide range of biological and cellular components of clones and donors. Although global methylation levels were similar, DMRs were identified, particularly in gene promoters, intergenic regions, and CpG islands near functionally critical genes. These DMRs were mapped to nearby genes, and the resulting DMGs were enriched for pathways related to mitochondrial function, energy metabolism, and cellular signaling. These epigenetic changes, though not linked to visible health issues in the clones, highlight incomplete reprogramming as a key area for improving cloning efficiency and stability. The findings of this study should be interpreted with caution due to the limited sample size. Additionally, further studies with larger samples are necessary to validate and strengthen these results.

## Figures and Tables

**Table 1:** Whole genome Nanopore PromethiON sequencing data.

Sample	Average Depth (X)	Total Reads (Number)	Mean Read Length (bp)	% Mapped Reads	Average Mapping Depth (X)
Donor 1	16.67	6,892,605	6288.9	99.90	16.05
Clone 1	16.54	6,337,029	6786.7	99.90	15.89
Donor 2	19.66	8,525,247	5995.8	99.84	18.52
Clone 2	17.96	7,132,741	6547.7	99.91	17.32

**Table 2:** Percentage distribution of identified genetic variants across different genomic regions in donor and cloned samples.

Genomic Region	Donor 1	Clone 1	Donor 2	Clone 2
DOWNSTREAM	5.0	5.1	6.9	5.0
INTERGENIC	18.8	19.9	14.5	18.8
INTRAGENIC	0.0	0.0	0.0	0.0
UPSTREAM	5.0	4.8	7.1	4.7
UTR_3_PRIME	0.7	0.7	1.0	0.6
EXON	1.3	1.2	2.0	1.2
INTRON	69.0	68.0	68.1	69.4
UTR_5_PRIME	0.22	0.21	0.4	0.2

**Table 3:** Distribution of structural variant types in donors and clones.

Variant Types	Donor 1	Clone 1	Donor 2	Clone 2
BND	1.44%	1.45%	1.63%	1.32%
DEL	60.50%	59.61%	60.31%	58.78%
DUP	0.32%	0.31%	0.30%	0.26%
INS	37.62%	38.52%	37.65%	39.55%
INV	0.09%	0.09%	0.09%	0.08%

Where BND = break ends; DEL = deletions; DUP = duplications; INS = insertions; INV = inversions.

**Table 4:** Gene biotype distribution of SVs in donors and clones.

Gene Biotype	Donor 1	Clone 1	Donor 2	Clone 2
Protein coding	86.248%	85.764%	86.759%	85.979%
lncRNA	11.580%	11.937%	11.167%	11.847%
pseudogene	2.030%	2.062%	1.866%	1.904%
C region (or Variable region)	0.041%	0.117%	0.117%	0.149%
Misc RNA	0.045%	0.058%	0.025%	0.059%
V segment	0.020%	0.034%	0.025%	0.032%
rRNA	0.010%	0.010%	0.006%	0.005%
snRNA	0.006%	0.006%	0.015%	0.011%
snoRNA	0.006%	0.003%	0.009%	0.005%
tRNA	0.006%	0.003%	0.006%	0.002%

**Table 5:** Total CpG count identified across the analyzed samples and top 100 most gene-level methylation count.

Animal	Total CPG	Top 100 Gene Methylation Count
Donor 1	9,408,361	402,837
Clone 1	9,319,431	400,398
Donor 2	10,131,221	455,049
Clone 2	9,963,519	428,795

**Table 6:** Total number of differentially methylated regions (DMRs), including counts of hypermethylated and hypomethylated regions across samples using a ≥10% methylation difference and a q-value threshold of 0.01.

	Total DMR Count	HyperMethylated Count	HypoMethylated Count	Significant Methylation Count (q-Value: 0.01)
Donor 1 vs. Clone 1	7,743,726	15	12	27
Donor 2 vs. Clone 2	8,821,206	38	103	141

In table (q-value < 0.01).

## Data Availability

All data generated or analyzed during this study is included in this published article.

## Supplementary Materials

The following supporting information can be downloaded at: https://www.mdpi.com/article/10.3390/ijms26188836/s1.

## Abbreviations

SCNT	Somatic cell nuclear transfer
ONT	Oxford Nanopore Technologies
WGS	Whole-genome sequencing
SMRT	PacBio’s single molecule technologies
SNV	Single nucleotide variants
SV	Structural variants
HMC	Handmade cloning
SNP	Single nucleotide polymorphism
Indels	Insertion and deletion
DMR	Differentially methylated regions
DEL	Deletions
INS	Insertion
BND	Breakends
INV	Inversion
NPR2	Natriuretic peptide receptor 2
chrMT	Chromosome of mitochondria
EDN1	Endothelin 1
NPPC	Natriuretic peptide C
EDNRA	Endothelin receptor type A
PGRMC1	Progesterone receptor membrane component 1
SPEM1	Spermatid maturation 1
APOE	Apolipoprotein E
XKR6	XK related 6
ATP8B1	ATPase phospholipid transporting 8B1
GO	Gene ontology
ND1	NADH dehydrogenase subunit 1
ND2	NADH dehydrogenase subunit 2
ATP6	ATP synthase F0 subunit 6
OR6Z9	Olfactory receptor family 6 subfamily Z member 9
OR9K15	Olfactory receptor family 9 subfamily K member 15
OR4C1F	Olfactory receptor family 4 subfamily C member 1F
mtDNA	Mitochondrial DNA
